# Supplementary material for: Monitoring the stability of transgene expression in lettuce using the RUBY reporter
Source: Plant Cell Rep. 2025 Nov 26;44(12):281. doi: 10.1007/s00299-025-03665-w (PMC12647185; doi:10.1007/s00299-025-03665-w)
Supplement: Supplementary file 3 — Supplementary file3 (DOCX 9 KB) [file 299_2025_3665_MOESM3_ESM.docx]

**Table S1. Constructs associated with this publication available in Addgene**

| **Plasmid Name** | **Description** | **Addgene Plasmid ID** |
| --- | --- | --- |
| pL0M-PU-pLsUBI | Level 0 GG module with the promoter from the Lactuca sativa polyubiquitin 4 gene. | 244448 |
| pL0M-T-tLsUBI | Level 0 GG module with the terminator from the Lactuca sativa polyubiquitin 4 gene. | 245484 |
| pL0M-SC-RUBY | Level 0 GG module with the RUBY coding sequence. | 245485 |
| pR2B5 | Level 1 transcription unit for pAtUBI-RUBY-tRBCS (R2 position in Golden Gate Level 2 Vector) | 245486 |
| pR2B6 | Level 1 transcription unit for pLsUBI-RUBY-tLsUBI (R2 position in Golden Gate Level 2 Vector) | 245487 |
| pATUBR | Binary plasmid with pAtUBI-RUBY-tRBCS for plant transformation (kanamycin plant selection marker) | 245488 |
| pLSUBR | Binary plasmid with pLsUBI-RUBY-tLsUBI for plant transformation (kanamycin plant selection marker) | 245489 |
| pJD849 | Binary plasmid with p35S-RUBY-tHSP for plant transformation (kanamycin plant selection marker) | 245490 |

**Table S2. Segregation Ratios of T_2_ families that were selected for propagation to the T_3_ generation.**

| **Construct** | **Line ID** | **Red** | **Rosy** | **Mixed** | **Green** |
| --- | --- | --- | --- | --- | --- |
| 35S:RUBY | 1 | 0 | 0 | 16 | 4 |
| 35S:RUBY | 2 | 2 | 0 | 8 | 4 |
| 35S:RUBY | 3 | 0 | 0 | 9 | 3 |
| 35S:RUBY | 4 | 0 | 0 | 6 | 4 |
| 35S:RUBY | 5 | 4 | 0 | 13 | 1 |
| AtUBI::RUBY | 6 | 4 | 4 | 1 | 5 |
| AtUBI::RUBY | 7 | 1 | 6 | 0 | 6 |
| AtUBI::RUBY | 8 | 2 | 10 | 2 | 2 |
| AtUBI::RUBY | 9 | 3 | 8 | 1 | 5 |
| LsUBI::RUBY | 10 | 9 | 0 | 0 | 1 |
| LsUBI::RUBY | 11 | 6 | 0 | 0 | 6 |
| LsUBI::RUBY | 12 | 9 | 0 | 0 | 2 |
| LsUBI::RUBY | 13 | 2 | 0 | 13 | 3 |
| LsUBI::RUBY | 14 | 11 | 0 | 0 | 3 |
| LsUBI::RUBY | 15 | 7 | 0 | 0 | 5 |
